# Evaluation of Educational YouTube Videos for Distal Radius Fracture Treatment

**DOI:** 10.1016/j.jhsg.2024.02.009

**Published:** 2024-03-25

**Authors:** Brandon S. Chai, Taewoong Chae, Adrian L. Huang

**Affiliations:** ∗ Faculty of Medicine, University of British Columbia, Vancouver, BC, Canada; † St. Paul’s Hospital Department of Orthopaedic Surgery, University of British Columbia, Vancouver, BC, Canada

**Keywords:** Distal radius fracture, Hand surgery, Social media, YouTube

## Abstract

**Purpose:**

Distal radius fractures (DRFs) are one of the most common fractures in adults. Adequate patient education is crucial for adherence to treatment. YouTube is a popular, accessible resource that has become a valuable tool for obtaining health information. The current study evaluated the top 50 YouTube videos on DRF treatment for patient education.

**Methods:**

A systematic search was conducted on YouTube using three searches to obtain 150 videos. Duplicate, nonrelevant, paid, and non-English videos were removed, and the top 50 rank-ordered videos were reviewed and characterized in terms of general (views, likes, video length, and publication date), source (publisher affiliation, presenter type, and target audience), and content (media type, topic coverage, advertisements, and bias) parameters.

**Results:**

Only 56% of videos were directed toward patients versus 40% for health care providers, highlighting a gap in patient-oriented educational content on YouTube. Most (86%) videos included effective visual aids, aligning with best practices for educational videos. Surgical management was overrepresented in 64% of the videos as opposed to nonsurgical management in 34% of videos. Only 31% of patient-oriented videos discussed surgical complications. Home exercises were emphasized in 75% of the videos discussing recovery topics.

**Conclusions:**

Although YouTube has the potential to be an effective resource for disseminating health information to patients, it has several limitations for education in DRF treatment including the lack of patient-oriented educational videos, overrepresentation of surgical treatment, and lack of information on surgical complications. Nonetheless, YouTube may have an important role as a supplementary resource, especially in certain topics such as guiding postoperative recovery with home exercises.

**Clinical relevance:**

This study allows health care providers and content creators to proactively address information gaps identified in educational YouTube videos on DRF treatment. It helps characterize the role of YouTube in supporting the treatment and recovery of patients experiencing DRFs.

Distal radius fractures (DRFs) are the most common fractures in adults, exhibiting a bimodal distribution with the highest incidence in younger men and older women.^1^ The incidence of DRFs is rising due to factors such as the aging population, increasing prevalence of childhood obesity, and rising popularity of e-scooters.^2^^,^^3^ These fractures can lead to significant functional impairment from reduced hand mobility if there is suboptimal treatment or rehabilitation.^4^ Consequently, the widespread incidence and morbidity of DRFs result in socioeconomic costs and loss of quality of life.^4^ Fortunately, patients sustaining DRFs generally have a good prognosis with limited functional impairment if they receive optimal treatment and adhere to rehabilitation.^2^ Therefore, proper patient education is essential to guiding adherence to treatment and rehabilitation for recovering hand function.

The internet has become an accessible, cost-efficient resource that has tremendously advanced health education for patients.^5^ It is an important resource with widespread dependence, as approximately 80% of internet users use the internet to seek health information.^5^ Although the internet offers a vast amount of health information, not all information is easy to consume—for example, many users endorse that text-based resources are more difficult to understand than video formats such as those offered by YouTube.^6^ YouTube is a popular video-streaming platform that is the second largest search engine after Google and is often a preferred resource for seeking information, especially in the younger population.^6^^,^^7^ However, there have been concerns about the quality and variability of medical and health-related informational videos on YouTube.^7^ Nonetheless, considering its accessibility, usability, and desired format for learning, YouTube has significant potential for delivering health information.

There are no prior studies investigating YouTube as an informational resource for DRF treatment and rehabilitation. Because sufficient patient education is necessary for adherence to treatment and rehabilitation, this study aims to evaluate YouTube videos on these topics. Although prior studies on YouTube videos for other orthopedic injuries focused on evaluating video quality, the primary objective of the current study was to identify key information gaps in DRF treatment to inform strategies for physicians to support these areas of patient understanding. It will aid health care providers in recommending resources for patients to provide ongoing support in their rehabilitation journey. Finally, it will advise future production of videos on DRF treatment and rehabilitation to address information gaps and adhere to best practices of video education.

## Methods

### Search strategy

Closed reduction and casting and surgery are the two treatment approaches for DRFs.^8^ As such, the search terms “Distal radius fracture treatment,” “Distal radius fracture surgery,” and “Distal radius fracture cast” were used to review the YouTube videos for DRF treatment on July 30, 2023. Data were programmatically web scraped (ie, extracted) from YouTube using a custom Python script (a custom code using Python programming language) that accessed search result data via YouTube Data API v3. The Python script systematically extracted data such as view count and publication date from each video retrieved through a search query. Subsequently, the results were exported to a comma-separated values file for further processing and analysis. The top 50 videos from each search query were recorded for a total of 150 videos.

### Screening

**Figure 1:**
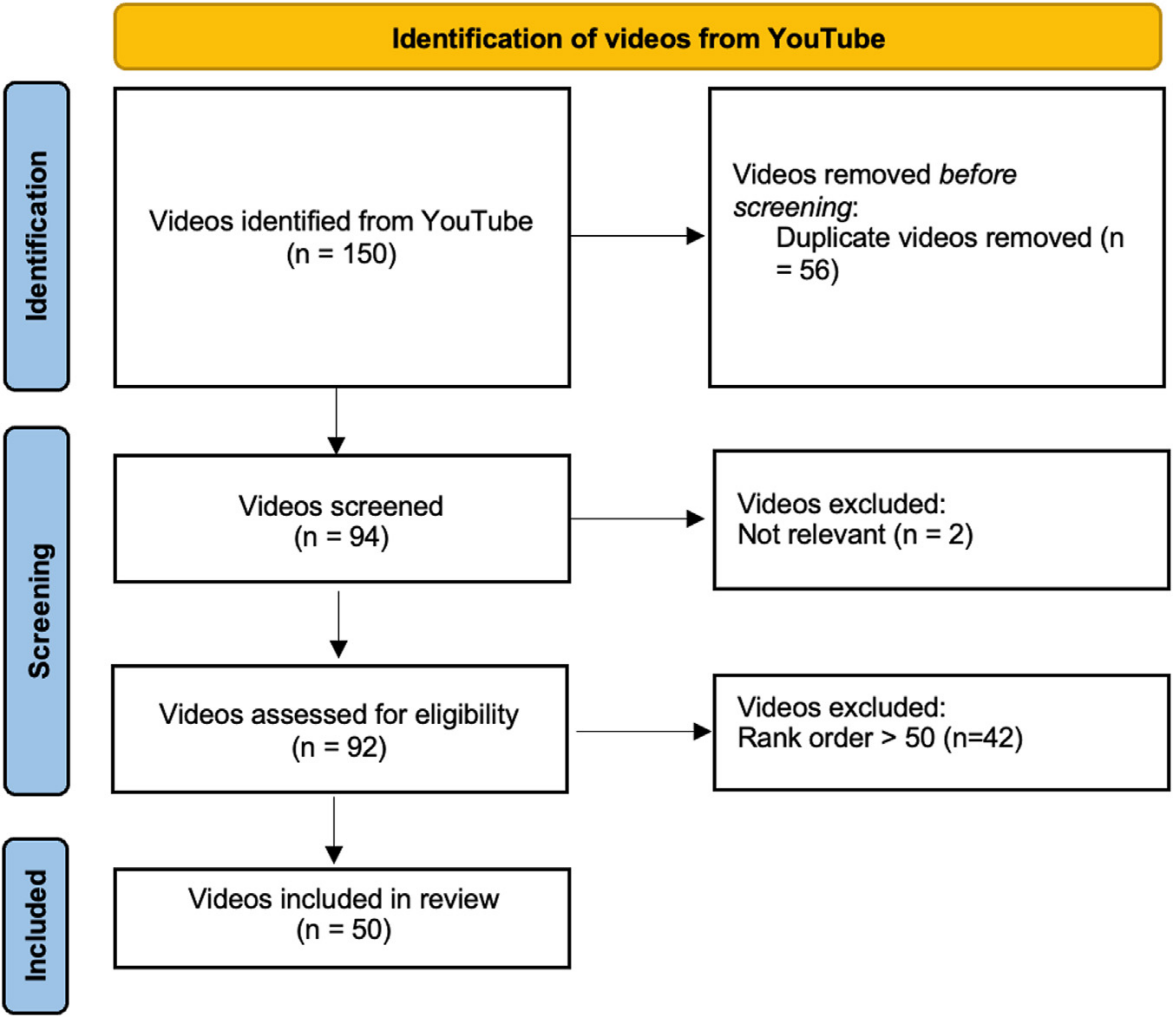
Modified PRISMA flow diagram depicting the systematic approach to screening and prioritizing YouTube videos on DRF treatment for full review.

The videos were screened as per the modified Preferred Reporting Items for Systematic Reviews and Meta-Analyses flow diagram (Fig. 1). Videos were rank-ordered based on the frequency of appearance between search lists and order of relevance. Duplicates were removed. Titles were screened using a lenient set of inclusion criteria as follows: videos must be (1) relevant to DRF treatment/rehabilitation, (2) available in English, and (3) free and accessible. The remaining videos comprising the final rank-ordered list were eligible for review.

Most patient clicks occur within the first 10 videos of a search list.^9^ Considering this, the top 50 videos on the final rank-ordered list were included in the review as these include the videos most likely to be viewed by patients.

### Data collection

There is not currently a gold standard tool for the evaluation of videos for health education.^10^ Although prior similar studies have used the Journal of the American Medical Association benchmark and DISCERN criteria to evaluate the quality of YouTube videos, these tools have significant limitations as these tools were created for the evaluation of online websites and written text, respectively.^10^ Consequently, evaluation of the quality of videos was not an objective of the study; therefore, these tools were not used in the evaluation of YouTube videos on DRF treatment.

The current study categorizes video data into general parameters (number of views, date of publication, number of likes, and video length), source parameters (country of origin, publisher affiliation, number of subscribers, and presenter type), and video content (topic coverage, media type, target audience, advertisements, number of comments, and gross bias) as described in previous studies.^11^^,^^12^ The goal of this review was to elucidate objective video characteristics and review the types of videos available for DRF treatment and rehabilitation; it is not a rigorous assessment of the video quality. As such, “gross bias” was defined as videos with blatantly incorrect or misleading information.

### Inter-rater reliability

A sample of 10 videos from each search were reviewed independently by three reviewers (AH, a hand surgeon with subspecialty training in upper extremity; BC, a medical student; and TC, a medical student). As data extraction from the videos was focused on high-level, patient-oriented information as opposed to detailed information for health care providers, advanced surgical knowledge was not deemed required for all reviewers. A protocol was established prior to review to address reviewer discordance. This included calculating kappa and intraclass coefficient statistics and iterative review of the videos. Video coding was discussed between the reviewers, resulting in high concordance with minimal discrepancies; hence, these coefficients were not calculated. The remaining 40 videos of each search were reviewed by one reviewer using a design-based research approach and iterative evaluation of the codes.^13^ Any remaining discrepancies were resolved via consensus with the other reviewer and/or a third independent reviewer.

### Statistical analysis

Descriptive statistics were used to represent quantitative data using Microsoft Excel.

## Results

### General parameters

The general parameters are summarized in Table 1. The number of views, duration, number of likes, and video age were represented by positive distributions. Nine videos were published within 2 years of the original searches, which is a guideline established for information currency.^14^

**Table 1:** General Parameters

Parameter	Mean (SD)	Median (Range)
View count	448,176 (2,332,704)	51,014 (911–16,570,498)
Like count	503 (712)	173 (0–2,600)
Video age (y)	5.2 y (3.4 y)	4.7 y (49 d–13.8 y)
Duration	8 min 58 s (14 min 14 s)	5 min 8 s (16 s–1 h 29 min 8 s)

### Source parameters

Table 2 displays characteristics related to the video source. Videos from personal accounts, a health care facility or organization, and commercial company were the most common publisher affiliations at 17 (34%), 15 (30%), and 11 (22%) videos, respectively. Twenty-seven videos (54%) included a physician as a presenter. This parameter was not mutually exclusive, and one video contained multiple presenters. Twenty-eight videos (56%) were targeted toward patients, with 20 (40%) videos for health care providers. Advertisements were present in nine (18%) videos. Subtitles were available in 43 (86%) videos. Gross bias was present in three (6%) videos (eg, a hand therapist declaring the necessity for prescribed therapy to achieve long-term functional recovery).

**Table 2:** Video Demographics

	Number of Videos (Percent of Total)
Country of origin	
USA	34 (68)
UK	6 (12)
India	3 (6)
Canada	1 (2)
Netherlands	1 (2)
Switzerland	1 (2)
Qatar	1 (2)
Unknown	3 (6)
Publisher affiliation	
Personal	17 (34)
Health Care Facility/Organization	15 (30)
Commercial	11 (22)
Nonprofit	3 (6)
Medical Journal	3 (6)
Educational Institution	1 (2)
Presenter type∗	
Physician	27 (54)
Hand therapist	5 (10)
Hand therapist	4 (8)
Patient	4 (8)
Physician assistant	3 (6)
Unknown	8 (16)
Target audience	
Patient	28 (56)
Health care provider	20 (40)
Both	2 (4)
Other	
Advertisements	9 (18)
English subtitles available	43 (86)
Gross bias	3 (6)

∗ Categories are not mutually exclusive.

### Video content

Lecture-style presentations (31% of videos; a presentation with or without slides as visual aids), interviews (34% of videos; presenter speaks directly into the camera with no visual aids), live surgeries (17% of videos; live footage of a surgical procedure), and computer animations (14% of videos; computer-generated images or videos) were common media types with roughly equal representation. Only two (4%) videos used live video, which consists of narrated “filler” footage of a patient’s experience during their care. Demonstrations (21% of videos) were a subset of the “Interview” format, which were videos with a presenter demonstrating exercises. Twenty (40%) videos had multiple media types, as this parameter was not mutually exclusive.

**Figure 2:**
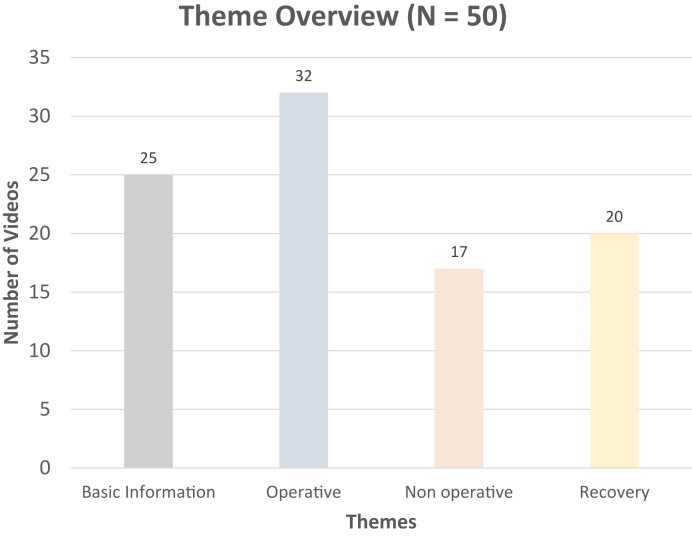
Distribution of the main themes identified in the YouTube videos for DRF treatment.

The following four main video content themes were identified, from most to least common: surgical management, basic information, recovery, and nonsurgical management (Fig. 2). Definitions of subthemes are found in the Supplementary Table 1 (available online on the Journal’s website at https://www.jhsgo.org).

**Figure 3:**
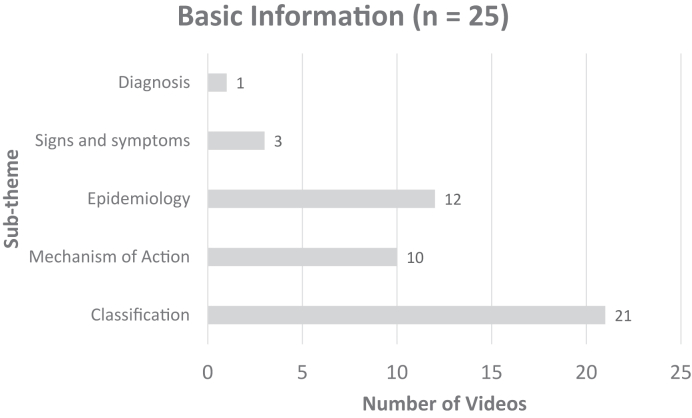
Distribution of the basic information subthemes for DRF treatment YouTube videos.

**Figure 4:**
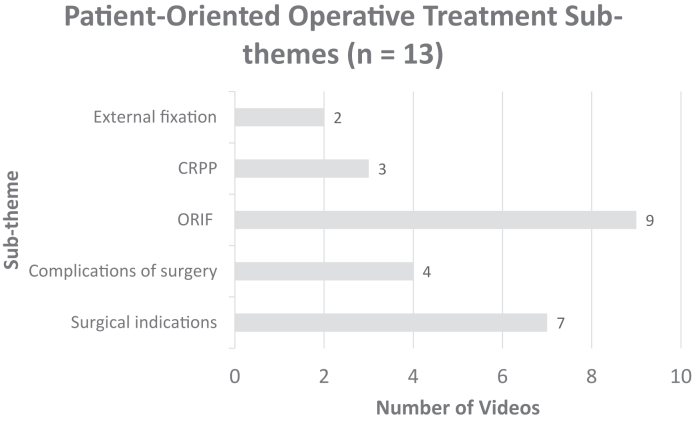
Distribution of surgical management subthemes for DRF treatment YouTube videos targeted toward patients. The distribution of subthemes excludes surgical management videos created for health care providers.

**Figure 5:**
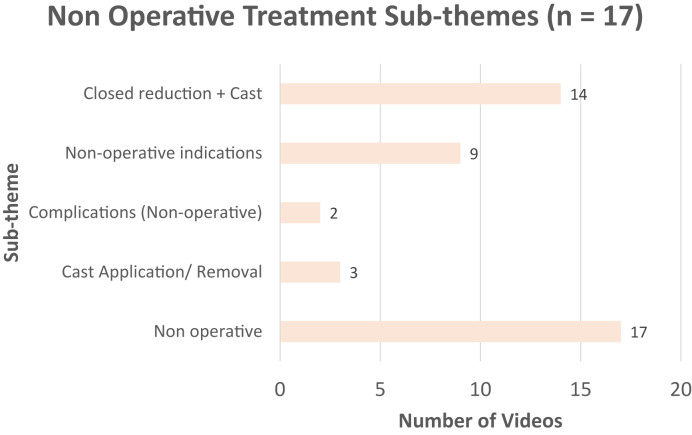
Distribution of nonsurgical treatment subthemes for DRF treatment YouTube videos.

**Figure 6:**
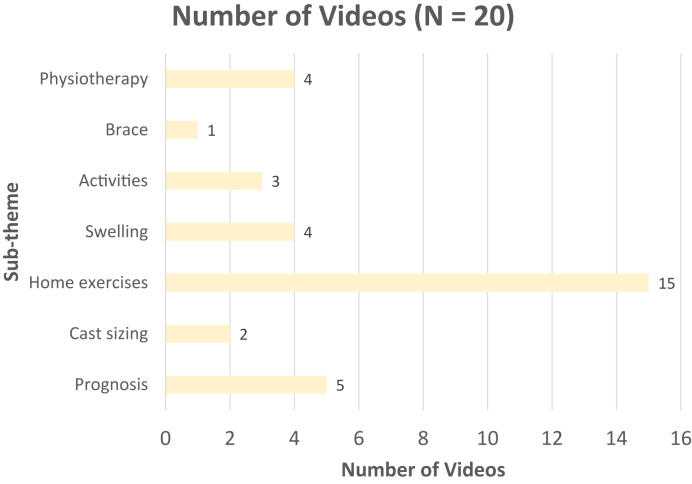
Distribution of recovery subthemes for DRF treatment YouTube videos.

Figure 3 displays the distribution of DRF basic information subthemes, with classification being the most common. There were 19 (59%) videos on surgical treatment that were made for health care providers and 13 (40%) videos that were oriented for patients. Videos with information on surgical management for health care providers discussed surgical treatment in advanced detail or presented a step-by-step live surgery to teach the viewer. The distribution of surgical treatment subthemes for patients is shown in Figure 4. The distribution of nonsurgical management and recovery subthemes are shown in Figures 5 and 6, respectively.

## Discussion

YouTube has become a common source for obtaining health information because of its minimal barriers to access and comprehensibility. The current study examined the top 50 YouTube videos on DRF treatment using a video rating tool that categorizes a variety of general, source, and content parameters.

Currency of health information is essential.^14^ Prior studies have demonstrated that medical information can quickly become outdated.^15^ For example, Shojania et al^16^ noted that 15% of systematic reviews become out-of-date within 1 year and 23% within 2 years.

In this study, only 9 (18%) of the videos were published within 2 years, which is a guideline for up-to-date information proposed by previous studies.^17^ As such, health care consumers should be aware that most YouTube videos on DRF treatment fall outside of this guideline and may potentially contain out-of-date information.

Videos were commonly published by personal YouTube accounts, which raises concerns about trustworthiness compared with professional sources such as health care facilities.^18^ Furthermore, health care consumers have a tendency to emphasize anecdotal information, especially from other patients, which can be misleading.^19^ However, 10 (59%) of the videos published by personal YouTube accounts were created by physicians, and only 2 (12%) by patients. Overall, physicians were the most common presenter type, regardless of affiliation. Taken altogether, these findings are reassuring as physician sources offer higher quality information and are preferred by patients than nonphysician sources.^20^^,^^21^ Future studies should identify factors of popular videos with high click-through rates to inform content creation and improve exposure to physician-made resources.

Only 56% of the videos targeted patients, whereas the remainder were for health care providers (40%) or both (4%). This is an interesting finding, as a greater proportion of patient-oriented videos was expected based on our layperson search terms of “treatment,” “surgery,” and “cast.” Perhaps this finding highlights a lack of patient-oriented information on DRF treatment on YouTube and is an area for content creators to further develop.

The dual channel processing theory proposes that both audio and effective visual (containing relevant visual aids such as images, animations, or demonstrations) channels are required for effective video learning.^22^^,^^23^ Forty-eight percent of the videos featured lecture-style presentations and live surgery media types, which were commonly used in health care provider–focused videos and aligned with this theory. Computer animations and live video are also media types that adhere to the dual channel theory principles, although more prevalent in patient-oriented videos. The interview was the most common media type and does not traditionally include effective visual aids as it typically follows a “talking head” format;^11^^,^^22^ however, most (60%) interview videos in the current study included demonstrations that were deemed to be effective visual aids, which complement the auditory component. Altogether, the vast majority (86%) of videos included salient visual aids that adhered to best practices for video education as per the dual channel processing theory.^11^

Approximately 80% of DRFs are treated nonoperatively.^1^^,^^24^ However, only 17 (34%) of the videos discussed nonsurgical treatment, whereas 32 (64%) videos focused on surgical treatment. This may overemphasize the role of surgical management in treating DRFs, possibly due to the perceived salience of surgery. Alternatively, there may be a greater need for educational resources in surgical than in nonsurgical management. This skewed representation may mislead viewers to perceive surgery as the more common approach rather than nonsurgical treatment. This finding highlights the importance for physicians to address these misconceptions during patient visits and encourages the production of videos on nonsurgical management to provide a more balanced understanding of DRF treatment.

Open reduction and internal fixation with plates and screws was the most common surgical technique discussed, aligning with standard surgical practices for DRFs.^22^^,^^23^ Closed reduction and percutaneous pinning and external fixation are less common approaches, which is reflected by the distribution.^8^^,^^25^ Surprisingly, only 31% of the patient-oriented videos covered surgical complications. This lack of information highlights the importance for surgeons to assess patients’ understanding of surgical risks and complications before obtaining informed consent. Nevertheless, content creators should aim to improve coverage of surgical complications to address this gap.

The role of home exercise programs versus hand therapy after DRF treatment remains inconclusive.^26^ Prior studies have demonstrated no difference in rehabilitation outcomes between these approaches for most patients.^26^ As such, a relatively balanced distribution of videos describing home exercises and hand therapy was expected. Interestingly, 75% of recovery videos focused on home exercises, whereas 20% on hand therapy. This emphasizes the need for more definitive evidence to support the role of home exercises after DRF treatment, given its prominent representation among recovery videos.

There are limitations to this study. The medical search term “distal radius fracture” may have biased the results toward videos oriented toward health care providers. Consequently, using a layperson term such as “broken wrist” may have yielded more patient-oriented videos but would have likely included common fracture patterns (eg, scaphoid) other than DRFs. Additionally, our search was only performed at one point in time, yielding a sample of videos representative of a snapshot in time. Finally, we did not assess the quality of these videos considering the lack of a gold standard tool for video evaluation.^10^ Until a gold standard tool is established, quality evaluation will continue to be challenging and limit studies evaluating YouTube for patient education.

The current study provides an overview of the general, source, and content parameters of the top 50 YouTube videos on DRF treatment. YouTube is a popular resource that has great potential for disseminating health information to patients; however, it is currently limited for education in DRF treatment, given the paucity of videos with a suitable target audience, overrepresentation of surgical management videos, and lack of information on surgical complications. These findings represent critical gaps in DRF treatment information on YouTube, which are essential for properly informing patients about their treatment options. As such, this study highlights an opportunity for physicians to address these knowledge gaps by producing quality YouTube videos in these areas. Additionally, it encourages physicians to proactively address misconceptions regarding nonsurgical versus surgical management for DRFs and discuss surgical complications (if applicable) during patient visits to further support patient education and improve care.

## Conflict of Interest

No benefits in any form have been received or will be received related directly to this article.

## Supplementary Data

e-Component
