# Supplementary material for: Evaluation of Educational YouTube Videos for Distal Radius Fracture Treatment
Source: J Hand Surg Glob Online. 2024 Mar 25;6(3):377–82. doi: 10.1016/j.jhsg.2024.02.009 (PMC11133890; doi:10.1016/j.jhsg.2024.02.009)
Supplement: e-Component [file mmc1.docx]

**Supplementary Data**

| Supplementary Table 1. Sub-theme Definitions | | |
| --- | --- | --- |
| Theme | Sub-theme | Definition |
| Basic Information | | |
|  | Anatomy and Classification of DRFs | Explains the anatomic structure involved and classification of DRFs with or without the use of radiographs. |
|  | Mechanism of Action (MoA) | Describes the MoA of DRFs. |
|  | Epidemiology | Describes the incidence of DRFs and populations most at risk. |
|  | Signs and Symptoms | Describes the signs and symptoms of DRFs. |
|  | Diagnosis | Describes the steps for diagnosing DRFs including physical exam and imaging. |
| Operative | | |
|  | Operative (healthcare providers) | Describes the specific steps for surgical repair or provides an advanced discussion on any operative sub-theme such as indications and complications. |
|  | External Fixation | Describes external fixation as a surgical treatment for DRFs at a patient level. |
|  | Closed Reduction Percutaneous Pinning (CRPP) | Describes CRPP as a surgical treatment for DRFs at a patient level. |
|  | Open Reduction Internal Fixation (ORIF) | Describes ORIF as a surgical treatment for DRFs at a patient level |
|  | Complications of Surgery | Describes potential complications of surgery such as irritation, hardware removal, tendon rupture or carpal tunnel syndrome at a patient level. |
|  | Surgical Indications | Discusses the general indications for surgery at a patient level. |
| Non Operative | | |
|  | Closed Reduction + Cast | Describes non-operative (closed reduction + cast) management, usually explained as the most common treatment option for DRFs. |
|  | Non-operative indications | Describes the general indications for non-operative management |
|  | Complications (Non-operative) | Describes the complications of non-operative management such as malunion. |
|  | Cast Application/ Removal | Demonstrates the process of cast application and/or removal. |
| Recovery | | |
|  | Physiotherapy | Describes the role of physiotherapy in recovery from DRFs. |
|  | Brace | Describes the use of a brace for support after removal of the cast. |
|  | Activities while in Cast | Encourages patients to continue participating in their daily activities and to use their injured hand as tolerated to help reduce stiffness. |
|  | Managing Swelling | Provides exercises specifically to reduce swelling including arm positioning (e.g. elevating arm above heart), massage and finger movements. |
|  | Home Exercises | Explains home exercises as essential in the recovery process to regain strength and range of movement, usually demonstrating specific exercises for patients. |
|  | Cast sizing | Discusses the importance of correct cast sizing and to contact the healthcare provider if cast is incorrectly sized. |
|  | Prognosis | Discusses the likelihood of returning to full range of movement and strength and/or the length of time for recovery. |
